# Serum antibodies against p53 in relation to cancer risk and prognosis in breast cancer: a population-based epidemiological study

**DOI:** 10.1038/sj.bjc.6690148

**Published:** 1999-02

**Authors:** P Lenner, F Wiklund, S O Emdin, C Arnerlöv, C Eklund, G Hallmans, H Zentgraf, J Dillner

**Affiliations:** Department of Oncology, Umeå University, Umeå, S-901 85 Umeå, Sweden; Department of Surgery, Umeå University, Umeå, S-901 85, Sweden; Microbiology and Tumor Biology Center, Karolinska Institute, Stockholm, S-17177, Sweden; Department of Pathology, Umeå University, Umeå, S-901 85, Sweden; Applied Tumor Virology, German Cancer Research Center, Heidelberg, D-69009, Germany

**Keywords:** breast cancer, p53, antibodies, case–control study, survival

## Abstract

To perform an epidemiological evaluation of the predictive value of p53 autoantibodies in breast cancer, we measured antibodies against p53 in serum samples from 165 breast cancer patients in comparison with serum samples from 330 healthy controls, selected from the same population as the cases and matched for age, sex and specimen storage time. Median age of patients was 51 years (range 25–64 years). Presence of serum p53 autoantibodies was analysed by enzyme-linked immunosorbent assay (ELISA) and confirmed by Western blotting. The lower ELISA reactivities were similar for cases and controls, but presence of high-level reactivity was more common among cases than among controls [odds ratio (OR) 9.03, 95% confidence interval (CI) 2.40–50.43]. Presence of Western blot-detected p53 autoantibodies had a very similar association (OR 10.8, CI 3.0–59.4). Among the cases, we also studied whether there was any correlation between level of anti-p53 antibodies and stage of the disease or survival. There was no significant correlation between presence of antibodies and stage of the disease. There was a significant negative correlation between presence of p53 antibodies and survival (*P*= 0.003). A stepwise multivariate Cox regression analysis showed that T-stage, age and presence of anti-p53 antibodies were significant independent prognostic variables, with a dose-dependent negative effect on survival for all three variables. We conclude that presence of anti-p53 antibodies are of significance both for the risk of having breast cancer and the risk of dying from breast cancer. *1999 Cancer Research Campaign*


*British Journal of Cancer* (1999) **79**(5/6), 927–932
© 1999 Cancer Research Campaign
Article no. bjoc.1998.0148

### Serum antibodies against p53 in relation to cancer risk and prognosis in breast cancer: a population-based epidemiological study

**P Lenner[1], F Wiklund[1], SO Emdin[2], C Arnerlöv[2], C Eklund[3], G Hallmans[4], H Zentgraf[5] and J Dillner[3]**

Departments of [1]Oncology and [2]Surgery, Umeå University, S-901 85 Umeå, Sweden; [3]Microbiology and Tumor Biology Center, Karolinska Institute, S-17177 Stockholm, Sweden; [4]Department of Pathology, Umeå University, S-901 85 Umeå, Sweden; [5]Applied Tumor Virology, German Cancer Research Center, D-69009 Heidelberg, Germany

**Summary** To perform an epidemiological evaluation of the predictive value of p53 autoantibodies in breast cancer, we measured antibodies against p53 in serum samples from 165 breast cancer patients in comparison with serum samples from 330 healthy controls, selected from the same population as the cases and matched for age, sex and specimen storage time. Median age of patients was 51 years (range 25–64 years). Presence of serum p53 autoantibodies was analysed by enzyme-linked immunosorbent assay (ELISA) and confirmed by Western blotting. The lower ELISA reactivities were similar for cases and controls, but presence of high-level reactivity was more common among cases than among controls [odds ratio (OR) 9.03, 95% confidence interval (CI) 2.40–50.43]. Presence of Western blot-detected p53 autoantibodies had a very similar association (OR 10.8, CI 3.0–59.4). Among the cases, we also studied whether there was any correlation between level of anti-p53 antibodies and stage of the disease or survival. There was no significant correlation between presence of antibodies and stage of the disease. There was a significant negative correlation between presence of p53 antibodies and survival ($P = 0.003$). A stepwise multivariate Cox regression analysis showed that T-stage, age and presence of anti-p53 antibodies were significant independent prognostic variables, with a dose-dependent negative effect on survival for all three variables. We conclude that presence of anti-p53 antibodies are of significance both for the risk of having breast cancer and the risk of dying from breast cancer.

**Keywords:** breast cancer; p53; antibodies; case–control study; survival

Alteration of the *p53* tumour-suppressor gene is the most common genetic change found so far in malignancy (Hollstein et al, 1991), including breast cancer (Osborne et al, 1991). The *p53* gene is of crucial importance for growth regulation, and it is therefore not surprising that *p53* alterations are consistently associated with more aggressive tumour biological factors and poorer prognosis in breast cancer (Davidoff et al, 1991*a*; Ostrowski et al, 1991; Hanzal et al, 1992; Isola et al, 1992; Thor et al, 1992; Allred et al, 1993*a,b*; Barnes et al, 1993; Noguchi et al, 1993; Silvestrini et al, 1993; Stenmark-Askmalm et al, 1994).

Autoantibodies against p53 have been detected in sera from breast cancer patients with prevalences ranging from 1% to 48% (Crawford et al, 1982; Davidoff et al, 1991*a*, 1992; Schlichtholz et al, 1992; Green et al, 1994; Mudenda et al, 1994; Porzolt et al, 1994; Lubin et al, 1995; Peyrat et al, 1995; Vojtesek et al, 1995; Coomber et al, 1996; Regidor et al, 1996; Willsher et al, 1996). In the study of Vojtesek et al 1995, which utilized three different methods to detect anti-p53 antibodies [immunoprecipitation of labelled protein, Western blotting and enzyme-linked immunosorbent assay (ELISA)], p53-specific circulating antibodies, confirmed by all methods, could be detected in only 1 of 100 cases (1%). With ELISA, 25% of the sera were considered positive, which was thought to be due to cross-reactions with unspecific immunogens. In the other studies, using ELISA-based methods, the reported frequency of positive sera has been in the range 12–48%. In most of these studies, the results were validated by immunoprecipitation or Western blotting.

Antibodies can be detected against mutated as well as wild-type p53 (Labrecque et al, 1993). Mutated p53 tends to have a much longer half-life than wild-type p53, leading to accumulation of the protein (Davidoff et al, 1991*b*). One study reported that there was a significant correlation between presence of p53 autoantibodies and the detection of mutated p53 protein in tissue sections (Mudenda et al, 1994). That study also reported a much higher detection rate of antibodies in patients with breast cancer than in normal control volunteers. There are also observations indicating that only tumours with missense mutations of p53, resulting in p53 overexpression, elicit p53 antibodies (Davidoff et al, 1992; Winter et al, 1992; Lubin et al, 1993; Houbiers et al, 1995). Anti-p53 antibodies have also been reported to indicate a poor prognosis in patients with breast cancer (Schlichtholz et al, 1992; Mudenda et al, 1994; Peyrat et al, 1995).

The objectives of the present study were to use an elaborate epidemiological approach to evaluate whether breast cancer patients do have an increased presence of p53 autoantibodies in comparison with matched controls selected from a population-based blood bank, and to investigate whether there was any correlation between antibody responses towards p53 and the stage at diagnosis as well as the survival of patients with breast cancer.

*Received 27 November 1997*
*Revised 11 May 1998*
*Accepted 29 July 1998*

*Correspondence to:* P Lenner, Department of Oncology, Umeå University, S-901 85 Umeå, Sweden

### MATERIALS AND METHODS

### Patients and controls

The case material consisted of 165 consecutive patients with newly diagnosed breast cancer admitted to the department of surgery at the University hospital of Umeå, between 1985 and 1992. Umeå University Hospital is the only hospital providing specialized breast cancer care for the population of the Västerbotten county in Northern Sweden. To obtain comparability with controls (see below), we excluded patients of age, at blood sampling, over 65 years. The age of the patients was thus 25–64 years (median 51 years). The tumours were clinically and pathologically staged using the TNM classification according to UICC (1982). In the majority of cases, treatment consisted of breast conserving surgery followed by radiotherapy of the breast and, in some cases, also the ipsilateral axilla. Patients with metastases in the axillary lymph nodes and/or stage T3 also received adjuvant therapy with tamoxifen or cytostatics. Patients with relapse or progression later in the course of disease were treated according to different protocols, mostly with cytostatics and/or hormonal regimens. Some patients received radiotherapy to metastatic sites. The patients were followed from date of diagnosis until death (from all causes), or to completion of follow-up (3 Nov 1995). Thus, the follow-up time was 1–120 months (median 73 months). No patient was lost to follow-up.

Pretreatment serum samples were, after informed consent, taken consecutively from all patients. The samples were frozen and stored at –80°C.

For each case ($n$ = 165), two controls were selected from the blood bank connected with the Västerbotten Project, which is a project offering health controls for the entire population of Västerbotten county in the age range 30–60 years. Currently, samples from about 50 000 subjects have been collected and stored at –80°C. The controls ($n$ = 330) were matched for gender and age at blood sampling (± 3 years). To assure storage times for cases and controls were as comparable as possible, we attempted to match also for time of sampling. For 55% of the triplets, this difference in sampling time between case and each of the corresponding two controls was less than 3 years, but for the rest of the material the difference was larger, up to 10 years at most.

### Serology

Levels of antibodies against p53 were analysed by ELISA using p53 protein, purified as described previously (Ryder et al, 1996). Analyses were performed with the analysing laboratory blinded to case–control status of the specimens. Cases and controls belonging to the same triplet were analysed together, minimizing the risk of bias due to possible interassay variations of the analysis procedure.

The p53 preparation used for ELISA assays was analysed by sodium dodecyl sulphate (SDS) polyacrylamide gel electrophoresis on a 10–15% gradient gel, followed by silver staining, to verify its purity and that it had not been degraded in transit. A major p53 band was detected (see Figure 1). p53 was coated onto ELISA plates (Costar, Cambridge, MA, USA) at 0.4 μg ml$^{-1}$ (the lowest coating concentration that gave maximal absorbance with reactive sera) in 0.1 M tris-HCl, pH 8.8, and incubated at 4°C overnight. After blocking with 10% horse serum in phosphate-buffered saline (PBS) for 1 h at 37°C, human sera [diluted 1:100 in 10% horse serum in PBS (HS-PBS)] was incubated on the plates for 2 h at 37°C. After washing with PBS/0.05% Tween 20 (PBS-T), a mouse monoclonal antibody to human IgG (gamma chain) (Eurodiagnostika, Malmö, Sweden), diluted 1:800 in HS-PBS, was incubated on the plates for 90 min at 37°C. After washing with PBS-T, a horseradish peroxidase conjugated antibody to mouse IgG (Southern Biotechnology) was incubated on the plates for 1 h at 37°C. After washing with PBS-T, the peroxidase substrate ABTS (Boehringer) was incubated on the plates for 1 h, whereafter the levels of absorbance were recorded at 405 nm.

For 96 sera (case–control triplets with two controls per case), the in-house optimized ELISA method was compared with a commercial p53 autoantibody detection kit (Dianova, Hamburg, Germany). The absorbances of reactive sera were similar by both methods.

**Table 1** Distribution of antibody levels against p53 among breast cancer patients and controls

| | Controls | | Cases | | | |
|---|---|---|---|---|---|---|
| p53 Cut-off | Number | % | Number | % | OR[a] | 95% CI[b] |
| 0–152 | 294 | 89 | 129 | 78 | 1.00 | |
| 153–291 | 33 | 10 | 24 | 15 | 1.88 | 0.96–3.69 |
| ≥292 | 3 | 1 | 12 | 7 | 9.03 | 2.40–50.43 |
| Total | 330 | 100 | 165 | 100 | | |

[a]Odds ratio; [b]confidence interval.

### Western blot

All serum samples with ELISA absorbances of 0.180 or more and some randomly chosen sera with lower reactivity, in total 48 serum samples, were also analysed for p53 reactivity by Western blot. Forty microgram per gel of p53 was run on 10–15% polyacrylamide gradient gels in a semiautomatic gel electrophoresis system (Phast system, Pharmacia, Uppsala, Sweden). The proteins of the gels were transferred to nitrocellulose sheets, which were cut into 15 strips per gel. The strips were blocked in 5% non-fat dry milk in PBS for 1 h, incubated with the human sera [diluted 1:100 in 2% non-fat dry milk in PBS (Whatman filtered)] for 2 h at room temperature, washed with 5% milk for 40 min with three changes of wash fluid, incubated with rabbit anti-human IgG-horseradish peroxidase conjugate (Dako, Copenhagen, Denmark) [diluted 1:1000 in 2% non-fat dry milk in PBS (Whatman filtered)] for 30 min at room temperature and then washed with PBS containing 0.5% Tween 20 for 2 h with ten changes of washing fluid. The strips were then developed using enhanced chemoluminescence as described by the manufacturer (Amersham-Pharmacia, Uppsala, Sweden).

### Statistical methods

Statistical analyses were carried out after division of antibody levels into three groups. The mean of the case mean and control mean was used as lower cut-off, and the mean of the controls plus two standard deviations as upper cut-off.

Multivariate conditional logistic regression for stratified data was used to calculate odds ratios with age at blood sampling, categorized into the three groups 25–49, 50–59 and 60–64 years, and

 © Cancer Research Campaign 1999

**Table 2** Distribution of antibody levels against p53 among cases of breast cancer for different T-, N- and M-stage

| | p53 | | | | | | | | |
|---|---|---|---|---|---|---|---|---|---|
| | 0–152 | | 153–291 | | ≥292 | | Total | | |
| | Number | % | Number | % | Number | % | Number | % | *P*-value[a] |
| T-stage[b] | | | | | | | | | |
| $T_1$ | 55 | 80 | 9 | 13 | 5 | 7 | 69 | 100 | |
| $T_2$ | 60 | 76 | 12 | 15 | 7 | 9 | 79 | 100 | |
| $T_3$ | 4 | 67 | 2 | 33 | 0 | 0 | 6 | 100 | |
| Total[c] | 119 | 77 | 23 | 15 | 12 | 8 | 154 | 100 | 0.50 |
| N-stage[b] | | | | | | | | | |
| N– | 47 | 82 | 5 | 9 | 5 | 9 | 57 | 100 | |
| N+ | 51 | 73 | 12 | 17 | 7 | 10 | 70 | 100 | |
| Total[c] | 98 | 77 | 17 | 13 | 12 | 9 | 127 | 100 | 0.26 |
| M-stage[b] | | | | | | | | | |
| M– | 86 | 81 | 12 | 11 | 8 | 8 | 106 | 100 | |
| M+ | 5 | 83 | 0 | 0 | 1 | 17 | 6 | 100 | |
| Total[c] | 91 | 81 | 12 | 11 | 9 | 8 | 112 | 100 | 1.00 |

[a]Jonckheere–Terpstra test; [b]pathologically staged; [c]based on total number of adequately investigated patients.

**Table 3** Multivariate Cox regression analysis. Lowest level chosen as reference for each variable

| Variable | RR[a] | 95% CI[b] |
|---|---|---|
| p53 | | |
| 0–152 | 1.00 | |
| 153–291 | 3.87 | 1.64–9.09 |
| ≥291 | 5.00 | 1.77–14.11 |
| T-stage[c] | | |
| $T_1$ | 1.00 | |
| $T_2$ | 5.05 | 1.90–13.37 |
| $T_3$ | 28.49 | 7.06–114.91 |
| Age at diagnosis | | |
| 25–49 | 1.00 | |
| 50–59 | 1.98 | 0.74–5.31 |
| 60–64 | 5.45 | 1.99–14.92 |

[a]Relative risk; [b]confidence interval; [c]pathologically staged.

antibody levels, categorized as described above, in the model. Exact confidence intervals for odds ratios were calculated using the permutational distribution of the sufficient statistics.

Associations between antibody levels and T-, N- and M-stage of the disease were tested with the Jonckheere–Terpstra test.

Kaplan–Meier estimate of survival and the log-rank test for trend was used to compare survival for different groups of antibody levels.

A multivariate Cox regression model was used to study prognosis in relation to antibody levels together with other prognostic factors. As explanatory variables, antibody level, categorized into three groups as explained above, T-stage ($T_1$, $T_2$ and $T_3$), N-stage (N– and N+), M-stage (M– and M+) and age at diagnosis, categorized into the three groups 25–49, 50–59 and 60–64 years, were used. Antibody level and age at diagnosis were included in the model as categorical variables with the lowest group as reference category. Backward stepwise analysis was used to select the final model.

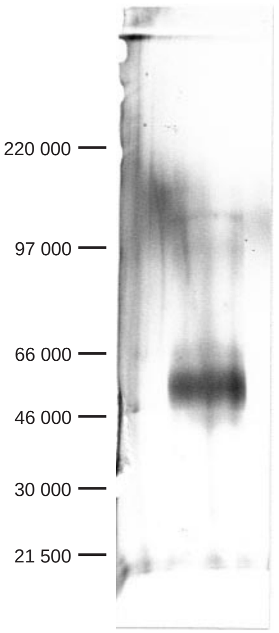

**Figure 1** Analysis of homogeneity of the p53 protein preparation by SDS-polyacrylamide gel electrophoresis and silver staining. About 200 ng of protein was applied to a 10–15% polyacrylamide gradient gel. The positions of the molecular weight markers are indicated on the left

### RESULTS

### Anti-p53 antibodies in relation to breast cancer risk

The distribution of antibody levels against p53 in cases and controls is shown in Table 1 and Figure 2. For cases and controls, the median absorbance values were 0.115 and 0.109, respectively, whereas the mean values were 0.198 and 0.106 respectively. The reason for the discrepancy between median and mean was the presence of a few patients with very high values. This skewness is

© Cancer Research Campaign 1999

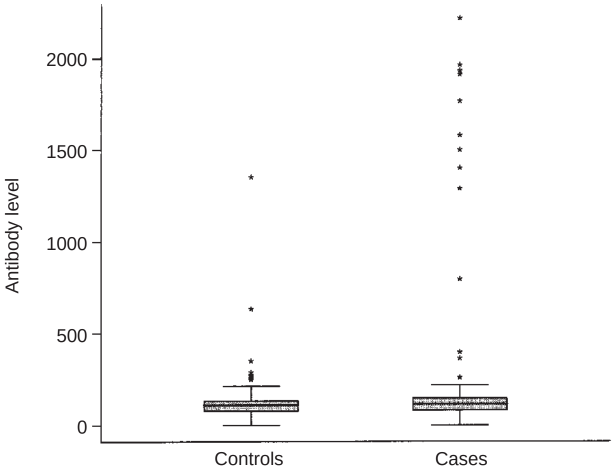

**Figure 2** Box plots showing the distribution of serum antibodies to p53 antigens for breast cancer patients and controls. The boundaries of the boxes represent 25% and 75% of the values, the lines in the boxes represent the median values and the lines connected to the boxes by vertical bars indicate the range of non-extreme values

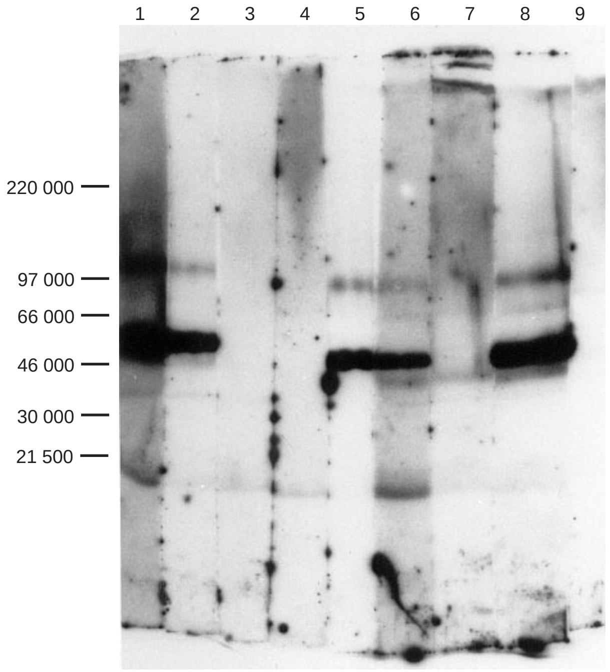

**Figure 3** Detection of serum antibodies to p53 by Western blotting. Nine nitrocellulose strips with the p53 protein preparation have been used to test nine different human sera for presence of serum IgG reactive with p53, as described in the Materials and methods section. The positions of the molecular weight markers are indicated on the left. Serum samples number 1, 2, 5, 6 and 8 were scored as p53 antibody positive, whereas serum samples 3, 4, 7 and 9 were scored as negative evident in the highest quartile, in which 30% of cases and 21% of controls were found. This discrepancy between cases and controls was, furthermore, confined to the highest values.

Lower and upper cut-off points for antibody levels were 0.152 and 0.291 respectively. In the logistic regression analysis, age at blood sampling did not have a significant effect, and removing it from the model yielded estimated odds ratios of 1.88 (95% confidence interval 0.96–3.69) and 9.03 (95% confidence interval

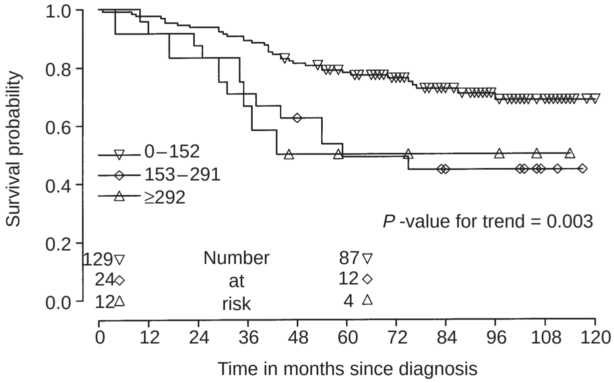

**Figure 4** Kaplan–Meier estimates of survival distributions for each group of antibody levels. Each mark on the curves represents a censoring time

2.40–50.43) for the middle and upper groups of p53 antibodies compared with the lowest group respectively (Table 1). Dividing the material at the lower cut-off point yielded an odds ratio of 2.60 (95% confidence interval 1.44–4.80) for those above compared with those under the cut-off value.

All sera with ELISA absorbances of 0.180 or more were also analysed by the confirmatory method, Western blotting (Figure 3). Most reactivities detected with the p53 band were very strong. Among the sera with ELISA absorbances above the high cut-off level (0.291), the reactivity was confirmed by Western blotting for all subjects except one. In the OD range 0.250–0.291, a p53 Western blot reactivity was detected in three out of six serum samples. In the OD range 0.180–0.249, a p53 Western blot reactivity was detected only in 1 out of 20 serum samples. The breast cancer risk associated with Western blot confirmed presence of p53 autoantibodies was similar to that of the ELISA high cut-off-detected p53 autoantibodies (3 out of 330 controls; 15 out of 165 cases; OR, 10.9; CI, 3.0–59.4).

Estimated odds ratios did not change significantly after excluding the 96 case–control triplets in which the age difference between case and control was more than 3 years, or the date of blood sampling differed by more than 3 years.

### Anti-p53 antibodies in relation to disease status

There was no significant correlation between serum level of p53 antibodies and T-, N- or M-stage of the disease (Table 2). Western blot reactivity was also not significantly associated with the T-, N- or M-stage of the disease (not shown).

### Anti-p53 antibodies in relation to prognosis

The univariate survival analysis was based on 165 cases, of which 54 were observed to die during follow-up. Survival was correlated with anti-p53 antibody level; the higher the antibody level, the shorter was the survival. Mean survival was 97, 73 and 71 months for the low, middle and upper group of antibody levels respectively. In Figure 4, the Kaplan–Meier estimate of the survival distributions is shown for each group of antibody levels. The log-rank test for trend showed a significant difference ($P$ = 0.003) in survival between the groups.

The multivariate survival analysis was based on 99 cases with complete data on all variables. Of these cases, 35 were observed to

*© Cancer Research Campaign 1999*

die during follow-up. The backward stepwise strategy in the multivariate Cox regression model with inclusion of stage (T-, N- and M-stage respectively), age at diagnosis and antibody level revealed that T-stage, age at diagnosis and antibody level remained as significant independent prognostic variables (Table 3). The relative risk estimates for different levels of the variables indicate the presence of a dose-dependent effect on survival for both level of anti-p53 antibodies and T-stage, besides age. Inclusion of Western blot-detected p53 autoantibodies instead of ELISA-detected p53 autoantibodies in the multivariate model also found that presence of anti-p53 autoantibodies had a significant independent effect on prognosis. The point estimate for the Western blot-detected p53 antibodies was 2.86 (CI, 1.13–7.26), which is somewhat lower than, albeit not statistically significantly different from, the prognostic value of presence of ELISA-detected p53 autoantibodies.

### DISCUSSION

The main finding of the present study was that a high serum level of antibodies directed against p53 was found more often in breast cancer cases than in age- and sex-matched normal subjects from the general population. In patients with breast cancer, presence of p53 autoantibodies also indicated a shortened survival. In this study, a population-based reference material was utilized, which is advantageous from an epidemiological point of view. To our knowledge, a population-based selection of controls has not been used in previous studies.

The finding of serum p53 autoantibodies in breast cancer is consistent with other studies. The first was Crawford et al (1982), who reported 9% p53-positive sera in 155 breast cancer patients by immunoprecipitation whereas none was positive among 164 healthy controls. Mudenda et al (1994) detected significantly higher antibody levels in breast cancer patients than in normal control volunteers with ELISA methodology. In the present study, population-based controls were used, matched for sex, age and time of specimen storage. The difference in anti-p53 antibody levels, measured with an ELISA-based method, was smaller between cases and controls in this study. Nevertheless, the difference was clearly evident for subjects with the highest absorbance levels of anti-p53 serum antibodies (absorbance values above 0.292), which correlated closely with the presence of p53 autoantibodies detectable also by Western blotting. Presence of ELISA reactivity that was not confirmed by Western blotting could be non-specific, directed against p53 conformational epitopes destroyed by denaturing in Western blotting or present in too low amounts to be detectable by the less sensitive Western blot technique. The fact that this low-level p53 ELISA reactivity (<0.297 absorbance) was significantly different from the reference group only in the prognostic analysis, but not in the case–control analysis, suggests a substantial unspecific component, the result of which is that a substantial positive predictive value of this low reactivity is only found in the high-prevalence population (the cases).

p53 in tumour tissue has been clearly demonstrated as a prognostic marker in breast cancer (Davidoff et al, 1991*a*; Ostrowski et al, 1991; Hanzal et al, 1992; Isola et al, 1992; Thor et al, 1992; Allred et al, 1993*a, b*; Barnes et al, 1993; Noguchi et al, 1993; Silvestrini et al, 1993; Stenmark-Askmalm et al, 1994). Increased p53 protein amounts in tumour cells are indicative of mutated *p53* (Davidoff et al, 1991*b*). An increase of p53 protein in tumours may elicit an immunological response (Winter et al, 1992), and therefore the finding of an association between serological anti-p53 response and survival in breast cancer is not surprising and has also been made by others (Peyrat et al, 1995). The multivariate analysis indicated that anti-p53 antibody level is an independent prognostic variable. The impact on survival by anti-p53 antibodies was not dependent on covariation with stage, which was also indicated by the lack of correlation between level of anti-p53 antibodies and clinical stage. This observation has also been made by other investigators (Schlichtholz et al, 1992; Peyrat et al, 1995), who found no significant correlation between p53 antibodies and clinical stage.

A question of great relevance from a theoretical and tumour biological point of view is when in the natural course of breast cancer the *p53* mutation is operative. In some cancers, including breast, alterations in *p53* have been shown to be an early event (reviewed in Lane et al, 1990). Anti-p53 antibodies have been detected before the diagnosis of breast cancer in 4 of 36 women with a positive history of breast cancer (Green et al, 1994). However, whether anti-p53 antibodies may be used as tumour markers in screening for breast cancer seems highly questionable.

In addition, the present study and others (Schlichtholz et al, 1992; Mudenda et al, 1994; Peyrat et al, 1995) demonstrate that presence of p53 autoantibodies is indicative of a worsened prognosis. Perhaps *p53* mutation could be viewed as an event that speeds up the disease development, and thus be regarded as a marker for a more aggressive behaviour of the tumour (Davidoff et al, 1991). It would, thus, be of interest to sample serum consecutively from breast cancer patients during the course of the disease, as well as sera taken before the diagnosis of breast cancer was made, and to correlate any p53 antibody elevation with the appearance of the disease, any recurrence of the disease, or other signs of aggravation of the disorder. Because the blood sampling and serological analysis of p53 antibodies are rather simple procedures, the methodology may have a potential as a prognostic marker in breast cancer in the future.

### ACKNOWLEDGEMENT

This study was supported by grants from the Research Foundation of the Department of Oncology, University of Umeå.

### REFERENCES

Allred DC, Clark GM, Elledge R, Fuqua SAW, Brown RW, Chamness GC, Osborne CK and Mcguire WL (1993*a*) Association of p53 protein expression with tumour cell proliferation rate and clinical outcome in node-negative breast cancer. *J Natl Cancer Inst* **85**: 200–206

Allred DC, Clark GM, Fuqua SAW, Elledge RM, Hilsenbeck SG, Ravdin PM, Yee D, Chamness GC and Osborne CK (1993*b*) Overexpression of p53 in node-positive breast cancer. *Breast Cancer Res Treat* **27**: 131

Barnes DM, Dublin EA, Fisher CJ, Levison DA and Millis RR (1993) Immunohistochemical detection of p53 protein in mammary carcinoma: an important new independent indicator of prognosis? *Hum Pathol* **24**: 469–476

Coomber D, Hawkins NJ, Clark M, Meagher A and Ward RL (1996) Characterisation and clinicopathological correlates of serum anti-p53 antibodies in breast and colon cancer. *J Cancer Res Clin Oncol* **122**: 757–762

Crawford LV, Pim DC and Bulbrook RD (1982) Detection of antibodies against the cellular protein p53 in sera from patients with breast cancer. *Int J Cancer* **30**: 403–408

Davidoff AM, Kerns BJM, Iglehart JD and Marks JR (1991*a*) Maintenance of p53 alterations throughout breast cancer progression. *Cancer Res* **51**: 2605–2610

Davidoff AM, Humphrey PA, Iglehart JD and Marks JR (1991*b*) Genetic basis for p53 overexpression in human breast cancer. *Proc Natl Acad Sci USA* **88**: 5006–5010

© Cancer Research Campaign 1999

Davidoff AM, Iglehart JD and Marks JR (1992) Immune response to p53 is dependent upon p53/HSP70 complexes in breast cancers. *Proc Natl Acad Sci USA* **89**: 3439–3442
Green JA, Mudenda B, Jenkins J, Leinster SJ, Tarunina M, Green B and Robertson L (1994) Serum p53 autoantibodies: incidence in familial breast cancer. *Eur J Cancer* **30A**: 580–584
Hanzal E, Gitsch G, Kohlberger P, Dadak CH, Miechowiecka N and Breitenecker G (1992) Immunohistochemical detection of mutant p53-suppressor gene product in patients with breast cancer: influence on metastasis-free survival. *Anticancer Res* **12**: 2325–2330
Hollstein M, Sidransky D, Vogelstein B and Harris C (1991) p53 mutations in human cancers. *Science* **253**: 252–254
Houbiers JGA, van der Burg SH, van de Watering LMG, Tollenaar RAEM, Brand A, van de Velde CJH and Melief CJM (1995) Antibodies against p53 are associated with poor prognosis of colorectal cancer. *Br J Cancer* **72**: 637–641
Isola J, Visakorpi T, Holli K and Kallioniemi O-P (1992) Association of overexpression of tumor suppressor protein p53 with rapid cell proliferation and poor prognosis in node-negative breast cancer patients. *J Natl Cancer Inst* **84**: 1109–1114
Labrecque S, Naor N, Thomson D and Matlashewski G (1993) Analysis of the anti-p53 antibody response in cancer patients. *Cancer Res* **53**: 3468–3471
Lane DP and Benchimol S (1990) p53: oncogene or anti-oncogene. *Genes Dev* **4**: 1–8
Lubin R, Schlichtholz B, Teillaud JL, Garay E, Bussel A, Wild CP and Soussi T (1995) p53 antibodies in patients with various types of cancer: assay, identification and characterization. *Clin Cancer Res* **1**: 1463–1469
Mudenda B, Green JA, Green B, Jenkins JR, Robertson L, Tarunina M and Leinster SJ (1994) The relationship between serum p53 autoantibodies and characteristics of human breast cancer. *Br J Cancer* **69**: 1115–1119
Noguchi M, Kitagawa H, Kinoshita K, Miyazaki I, Saito Y and Mizukami Y (1993) Prognostic significance of p53 and c-erbB-2 experience in operable breast cancer. *Int J Oncol (Greece)* **2**: 587–591
Osborne RJ, Merlo GR, Mitsudomi T, Venesio T, Liscia DS, Cappa APM, Chiba I, Takahashi T, Nau MM, Callahan R and Minna JD (1991) Mutations in the p53 gene in primary human breast cancers. *Cancer Res* **51**: 6194–6198
Ostrowski JL, Sawan A, Henry L, Wright C, Henry JA, Hennessy C, Lennard JW, Angus B and Horne HW (1991) p53 expression in human breast cancer related to survival and prognostic factors: an immunohistochemical study. *J Pathol* **164**: 75–81
Peyrat JP, Bonneterre J, Lubin R, Vanlemmens L, Fournier J and Soussi T (1995) Prognostic significance of circulating p53 antibodies in patients undergoing surgery for locoregional breast cancer. *Lancet* **345**: 621–622
Porzolt FMS, Hoher D, Muche D, Gaus W and Montenarth M (1994) Biological relevance of auto-antibodies against p53 in patients with metastatic breast cancer. *Onkologie* **17**: 402–408
Regidor PA, Regidor M, Callies R and Schindler AE (1996) Detection of p53 auto-antibodies in the sera of breast cancer patients with a new recurrence using an ELISA assay. Does a correlation with the recurrence free interval exist? *Eur J Gyanaecol Oncol* **17**: 192–199
Ryder SD, Rizzi PM, Volkmann M, Metivier E, Pereira LM, Galle PR, Naoumov NV, Zentgraf H and Williams R (1996) Use of specific ELISA for the detection of antibodies directed against p53 protein in patients with hepatocellular carcinoma. *J Clin Pathol* **49**: 295–299
Schlichtholz B, Legros Y and Gillet D (1992) The immune response to p53 in breast cancer patients is directed against immunodominant epitopes unrelated to the mutational hot spot. *Cancer Res* **52**: 6380–6384
Silvestrini R, Benini E, Daidone MG, Veneroni S, Boracchi P, Cappelletti V, Di Fronzo G and Veronesi U (1993) p53 as an independent prognostic marker in lymph node-negative breast cancer patients. *J Natl Cancer Inst* **85**: 965–970
Stenmark-Askmalm M, Stål O, Sulliwan S, Ferraud L, Xiao-Feng S, Carstensen J and Nordenskjöld B (1994) Cellular accumulation of p53 protein: an independent prognostic factor in stage II breast cancer. *Eur J Cancer* **30A**: 175–180
Thor AD, Moore DHI, Edgerton SM, Kawasaki ES, Reihsaus E, Lynch HT, Marcus JN, Schwartz L, Chen L-C, Mayall BH and Smith HS (1992) Accumulation of p53 tumor suppressor gene protein: an independent marker of prognosis in breast cancer. *J Natl Cancer Inst* **84**: 845–855
UICC (1982) *TNM Classification of Malignant Tumours,* 3rd edn. Springer Verlag, Geneva
Vojtesek B, Kovarik J, Dolezalova H, Nenutil R, Havlis P, Brentani RR and Lane DP (1995) Absence of p53 autoantibodies in a significant proportion of breast cancer patients. *Br J Cancer* **71**: 1253–1256
Willsher PC, Pinder SE, Robertson L, Nicholson RI, Ellis IO, Bell JA, Blamey RW, Green JA and Robertson JF (1996) The significance of p53 autoantibodies in the serum of patients with breast cancer. *Anticancer Res* **16**: 927–930
Winter SF, Minna JD, Johnson BE, Takahashi T, Gazdar AF and Carbone DP (1992) Development of antibodies against p53 in lung cancer patients appears to be dependent on the type of p53 mutation. *Cancer Res* **52**: 4168–4174

*© Cancer Research Campaign 1999*
